# Ancient CO_2_ levels favor nitrogen fixing plants over a broader range of soil N compared to present

**DOI:** 10.1038/s41598-021-82701-7

**Published:** 2021-02-04

**Authors:** Haoran Chen, John Markham

**Affiliations:** Department of Biological Sciences, University of Manitoba, Winnipeg, Canada

**Keywords:** Plant sciences, Ecology

## Abstract

Small inreases in CO_2_ stimulate nitrogen fixation and plant growth. Increasing soil N can inhibit nitrogen fixation. However, no studies to date have tested how nitrogen fixing plants perform under ancient CO_2_ levels (100 MYA), when nitrogen fixing plants evolved, with different levels of N additions. The aim of this study was to assess if ancient CO_2_, compared to present, favors nitrogen fixers over a range of soil nitrogen concentrations. Nitrogen fixers (*Alnus incana* ssp. *rugosa*, *Alnus viridis* ssp. *crispa*, and *Alnus rubra*) and their close non-nitrogen fixing relatives (*Betula pumila*, *Betula papyrifera*, *Betula glandulosa*) were grown at ancient (1600 ppm) or present (400 ppm) CO_2_ over a range of soil N levels, equivalent to 0, 10, 50, and 200 kg N ha^−1^ year^−1^. The growth of non-N fixing plants increased more than N fixing plants in response to the increasing N levels. When grown at an ancient CO_2_ level, the N level at which non-nitrogen fixing plant biomass exceeded nitrogen fixing plant biomass was twice as high (61 kg N ha^−1^ year^−1^) as the N level when plants were grown at the ambient CO_2_ level. Specific nodule activity was also reduced with an increasing level of soil N. Our results show there was a greater advantage in being a nitrogen fixer under ancient levels of CO_2_ compared with the present CO_2_ level.

## Introduction

Fossil evidence shows that nitrogen fixing plants first evolved in the late Cretaceous, when atmospheric CO_2_ was *ca.* four times higher than at present^1,2^. Although there is some question as to whether the evolution of symbiotic nitrogen fixation arose once, or multiple times, in a small number of related clades^3^, it is clear that symbiotic nitrogen fixation is a trait that has been lost repeatedly. This has resulted in most taxa within the nitrogen fixing clade lacking the ability to fix nitrogen^4^. It has been suggested that the decrease in CO_2_ levels over geological time provided the global selection pressure to allow for this loss of the nitrogen fixing trait across the entire nitrogen fixing clade^3^.

There is also, at present, a low abundance of nitrogen fixing plants in many parts of the globe^5^. Symbiotic nitrogen fixing plant species abundance, and to a lesser degree species richness, decreases with latitude, with legume species having a higher abundance at lower latitudes (< 35º), and actinorhizal species having a higher abundance at higher latitudes^6,7^. The actinorhizal plants are mainly pioneer species, colonizing N poor soils, where they have access to high light intensity in the early stages of succession^8^. One reason for the present lack of success of nitrogen fixing plants may relate to the high energy cost of nitrogen fixation, especially at higher latitudes^9^. In all but N poor soils, nitrogen fixing plant plants spend more energy in acquiring nitrogen from fixation than non-N fixers taking up soil inorganic N. In addition, N fixers indirectly provide fixed nitrogen to their non-N fixing competitors, which may then shade them^8^. Menge and Crews^5^ have also argued that actinorhizal plants are obligate N fixers (unlike legumes), and thus cannot down-regulate symbiotic nitrogen fixation. There are however a lack of studies comparing the performance of N-fixers to non-N fixers, especially to closely related non-N fixers with the same growth form, under environmental conditions that are predicted to alter their performance.

The loss of the nitrogen fixing trait with decreasing CO_2_ levels over geological time may relate to the energetic cost of fixation, with decreased CO_2_ limiting energy capture, and to plants ability to balance carbon and nitrogen acquisition. The recent industrial era increases in atmospheric CO_2_ show that increasing CO_2_ increases plant N demand in two ways. First, increasing CO_2_ concentration increases C sequestration resulting in a simultaneous increase in N uptake from the soil^10^. Secondly, increased CO_2_ levels increase the C:N ratio of leaves, and consequently the C:N ratio of the soil, which decreases soil N availability^10,11^. Elevated CO_2_ therefore increases both symbiotic and free living nitrogen fixation compared with ambient CO_2_ levels^11,12^. Although many studies have focused on the effect of increasing atmospheric CO_2_ on biological nitrogen fixation, they have been restricted to less than 800 ppm CO_2_ levels in order to understand future scenarios^11,12^. There is no study comparing nitrogen fixing plant growth at ambient and ancient CO_2_ levels.

Another factor that may negatively affect nitrogen fixing plants, especially obligate N fixers, is increasing N deposition. Low levels of available N can critically limit the establishment and growth of non-N fixers. In such N-poor areas, actinorhizal plants are favored^13^. At present, human activities have resulted in increased N deposition at a global scale. N deposition increases have arisen from the unintentional production of NO_x_ in the atmosphere and the intentional production of reduced N (NH_x_) for synthetic fertilizer^14^. The global rate of anthropogenic N inputs has increased from *ca.* 100 Tg N year^−1^ to *ca.* 200 Tg N year^−1^ during the past 20 years^15–17^. Currently, 11% of natural vegetation in North America, Europe, south and east Asia receives anthropogenic atmospheric nitrogen deposition of over 10 kg N ha^−1^ year^−1^, a level that can have significant effects on ecosystem processes^14^. The annual total N deposition in some areas is projected to further increase in the future, ranging from 20 to 50 kg N ha^−1^ year^−1^ by 2100^14,18^. Since the net production of many terrestrial ecosystems is limited by N availability, N deposition should enhance ecosystem productivity. On the other hand, N deposition can threaten soil quality through soil acidification, altering the ground flora, leading to a decline of productivity and biodiversity loss in some ecosystems^19,20^. Nitrogen deposition has been repeatedly reported to reduce biodiversity in some ecosystems^21,22^. Global rates of biological nitrogen fixation have also fallen from over 100 Tg year^−1^ to *ca.* 44 Tg year^−1^ from the 1990s to 2013^15,23^, which is largely due to anthropogenic N inputs^16,23^. It has also been reported that plants confined to growing in low N soils and those depending on N fixation are more negatively affected by increased N deposition^21^. It was found that N fixing plants are more susceptible to declines in performance than non-nitrogen fixing plants, with increased N deposition, due to a reduced competitive ability^24^. Some nitrogen fixers are able to adjust to soil N additions by reducing nitrogen fixation. For example, a 2-year field study found that 180 kg N ha^−1^ application reduced biological nitrogen fixation of soybean (*Glycine max* (L.) Merr) compared with non-fertilizer treatments^25^. While it has been argued that actinorhizal plants cannot down-regulate symbiotic nitrogen fixation^5^, lab studies have shown that actinorhizal plants can shift from using fixed-N to using N-derived fertilizer with an increasing level of N fertilization^26^. This may help them to cope with increased N deposition.

We know little about the effects of ancient levels of CO_2_ and soil nitrogen levels on the relative performance of nitrogen fixing and non-nitrogen fixing plants. We know elevated CO_2_ leads to the increase of plant C:N ratio across all plants^27–29^. Therefore, increases in N deposition could potentially buffer the effect increases in atmosphere CO_2_ on plant tissue C:N ratio^30^.

The clade that includes actinorhizal plants also contains non-nitrogen fixing species. This makes them ideal for comparing species with and without the ability to form a nitrogen fixing symbiosis. The purpose of this study was to look at the effect of soil nitrogen levels on nitrogen fixing plants under present and ancient levels atmospheric CO_2_ levels by comparing them to their close non-nitrogen fixing relatives. We hypothesized nitrogen fixing plants will maintain greater growth with increasing N levels under ancient CO_2_ conditions compared with present, helping to explain the evolution of symbiotic nitrogen fixation in the Late Cretaceous. We also hypothesized that actinorhizal N-fixing plants are partially facultative N-fixers and so can downregulate N fixation when N deposition is high at all level of CO_2_.

## Results

**Figure 1:**
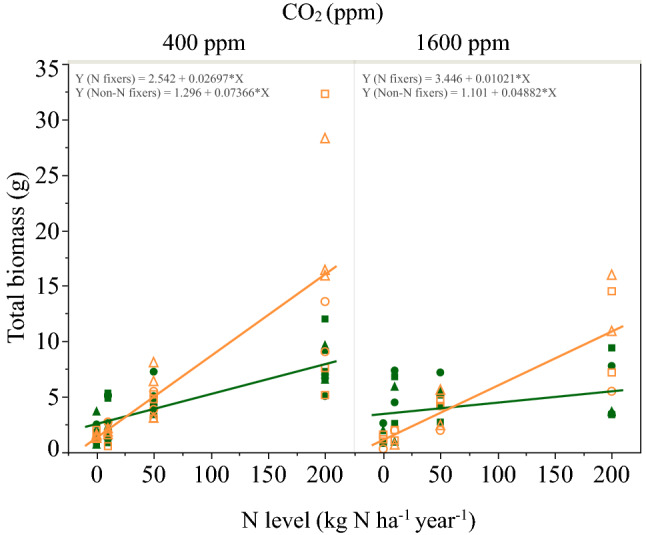
The response of nitrogen fixing (green, solid symbols) and non-N fixing (orange, open symbols) plant groups to the increasing soil N level at 400 and 1600 ppm CO_2_ (P = 0.006, F = 8.03 for the interaction between N and CO_2_ level; P < 0.0001, F = 28.22 for the interaction between the plant group and nitrogen level). Species symbols: *A. rugosa*—green round, *A. rubra*—green triangle, *A. crispa*—green square, *B. glandulosa*—orange round, *B. papyrifera*—orange triangle, *B. pumila*—orange square.

The effect of nitrogen on plant mass varied with CO_2_ level (P = 0.006, F = 8.03 for the nitrogen by CO_2_ level interaction in the least squares model), and differed between the nitrogen fixing and non-nitrogen fixing plants (P < 0.0001, F = 28.22 for the plant group by nitrogen level interaction), but the model showed no effect of species within plant groups (P = 0.20, F = 1.55, Supplementary Figure S1). We therefore performed a least square fits of the responses of nitrogen fixing and non-nitrogen fixing plants to soil nitrogen additions at each CO_2_ level separately (Fig. 1, Supplementary Table S1). At both ambient and ancient CO_2_ levels, non-nitrogen fixing plant mass increased faster than nitrogen fixing plants mass in response to N addition. However, because there was a greater difference in the rate of plant mass increase between the plant types at 400 ppm than at 1600 ppm CO_2_, the N level at which the two plant groups performed equally well (i.e., the mass of plants was equal) differed between the two CO_2_ levels. At 400 ppm CO_2_, plant biomass of the nitrogen fixing and non-nitrogen fixing plants was equal at a soil N deposition rate of 27 kg N ha^−1^ year^−1^, while at 1600 ppm CO_2_, the N level at which plant biomass was equal was at 61 kg N ha^−1^ year^−1^, more than double the N when plants were grown at 400 ppm CO_2_.

**Figure 2:**
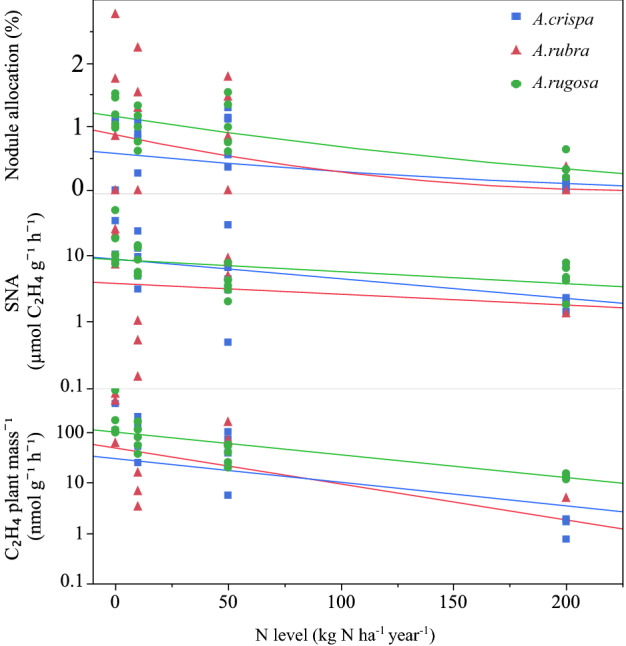
The effect of soil N level on the proportion biomass allocated to nodules, acetylene reduced per nodule mass (SNA) and per plant mass.

Increasing soil N reduced all parameters associated with nitrogen fixation, regardless of CO_2_ level, with significant differences between plant species for some parameters (Fig. 2, Supplementary Table S2). Biomass allocation to nodules, including plants that failed to form nodules, decreased from 0.98 ± 0.21% (± standard error) of total biomass at no N addition to 0.17 ± 0.05% at an addition rate of 200 kg N ha^−1^ year^−1^ (P < 0.0001, F = 20.99 for the N addition effect on square root transformed data), with a lower allocation for *A*. *viridis* spp. *crispa* than for *A*. *incana* spp. *rugosa* (P = 0.04, F = 3.35 for the species effect). Also, at the 200 kg N ha^−1^ year^−1^ addition rate, 33% of the plants failed to form nodules, whereas only 16% of the plants failed to form nodule at all other nitrogen addition levels combined. An ANOVA model using only plants grown at this highest N addition rate indicated that there was no difference in plant mass between plants that formed nodules and plants that did not (P = 0.61, F = 0.27). For the plants with nodules, specific nodule activity (SNA) decreased from 18.48 ± 4.10 µmol C_2_H_4_ g^−1^ h^−1^ at no N addition to 3.85 ± 0.77 µmol C_2_H_4_ g^−1^ h^−1^ at a N addition rate of 200 kg N ha^−1^ year^−1^ (P = 0.02, F = 5.61 for the N addition effect on log transformed data) with no differences between the three species. Because both allocation to nodules and their physiological activity declined with increases N addition, there was an overall reduction in nitrogenase activity per plant mass from193.57 ± 60.67 nmol C_2_H_4_ g^−1^ h^−1^ at no N addition to 6.04 ± 1.68 nmol C_2_H_4_ g^−1^ h^−1^ at a N addition rate of 200 kg ha^−1^ year^−1^ (P < 0.0001, F = 20.98 for the N addition effect on log transformed data) with *A. viridis* spp. *crispa* having significantly lower rates than A. *incana* spp. *rugosa* (P = 0.03, F = 3.64).

## Discussion

Nitrogen is the primary limiting nutrient for plant growth. Our study found that increasing N deposition did not bring as great benefits to N-fixers (*Alnus* spp.) compared with closely related non-N fixers (*Betula* spp.) in two ways. First, we found that biomass production of non-N fixing plants responded more strongly to increasing levels of soil N at both ambient and ancient CO_2_, compared with N fixing plants. N fixing plants grew more than non-N fixing plants at low levels of soil N, while high levels of soil N resulted in greater increases in growth of non-N fixing plants. Our results support the previous studies suggesting that N fertilization has limited effect on N fixing plant growth but increases the growth and abundance of unrelated non-N fixers^31–34^. For example, Skogen^24^ found the ratio of aboveground mass of a non-fixer (*Solidago canadensis*) to an N fixer (*Desmodium cuspidatum*) increased with the increasing levels of N deposition. This suggested that N fixing plants are not competitive under high level of N when they grow with non-N fixers. Our study showed that under present atmospheric CO_2_ conditions N-fixing plants will only have an advantage over non-N fixing plants at low levels of soil N. Second, our study found that increasing levels of N decrease nodule allocation, specific nodule activity and nitrogen fixation rate per plant mass. This shows that actinorhizal plants can be facultative N fixers, downregulating N fixation at high levels of soil N, as has been found in other lab studies^26^. This would seem to contradict Menge and Crews^5^ hypothesis that actinorhizal plants are unable to maintain their dominance in high latitude regions because they are unable to switch from nitrogen fixation to inorganic soil N uptake, and so they become less competitive as soil N levels increase. We agree that under most conditions where actinorhizal plants are found, there is little regulation of nitrogen fixation since soil N levels are not high enough to affect a response in the plants. More importantly, even though actinorhizal plants can reduce nodule formation and activity as soil N increases, they are not able to increase growth to the same degree that closely related non-nitrogen fixing plants can, making them less competitive.

Atmospheric CO_2_ levels have been decreasing since nitrogen fixing plants first evolved. Our study suggests there is a greater advantage in being a nitrogen fixer under ancient levels of CO_2_, in that it takes a higher level of soil N for non-nitrogen fixing plants to have greater growth than nitrogen fixing plants. This implies that there was greater selection pressure in the past for the evolution of symbiotic nitrogen fixation. Our results support the hypothesis that the decreasing CO_2_ constrains favoring the evolution of symbiotic plant nitrogen fixation^3^. At present the clade of plants that includes the actinorhizal plants has many non-nitrogen fixing species as well^35,36^. It is likely that the ability to fix nitrogen has been lost numerous times since it first evolved^2,4,37^. As atmospheric CO_2_ levels dropped since the Late Cretaceous, there is likely to have been increasing selective pressure to lose the ability to fix nitrogen but utilize nitrogen from the soil^3^. Under current rising CO_2_ levels, there is some evidence that nitrogen fixation is becoming a more favorable trait, at least in the absence of other nutrient limitations (e.g., P, Mo)^12,38^. At the same time, anthropogenic inputs of N tend to counteract this advantage of being a nitrogen fixer^39,40^.

Our study brings a new sight into why nitrogen-fixing plants are not more ubiquitous at present. Under ancient CO_2_ levels, plants likely had a higher demand for N to maintain their C:N ratio^41^. These conditions would have likely facilitated the evolution of nodulation to meet plants high N requirement^3,5^. As global CO_2_ decreased in the past 100 million years, there would be less of a demand for N uptake. Consequently, N fixers would be at a competitive disadvantage to non-N fixers, which would select for the loss of nodulation that is evdident in the N-fixing clade of plants. It is unclear from our data why nitrogen-fixing plants do not have the same rate of growth increase in response to nitrogen additions as non-nitrogen fixing plants do, at both ambient and ancient CO_2_ levels. One possibility is that while increased N availability results nitrogen fixing plants down-regulating their N fixation rate, they still allocate carbon for nodule maintenance and growth. Skogen^24^ has also suggested that nitrogen-fixing plants cannot uptake inorganic N as efficiently as non-N fixing plants, but the mechanism for this has not been described. So the high energy cost for nodule maintenance and a low N use efficiency might explain that N-fixing plants grow slower than non-N fixing plants in response to the increasing N levels.

Our study did not show a stimulation of nitrogen fixation and plant growth at an ancient CO_2_ level. Previous study has shown that stimulation of N fixation under elevated CO_2_ is limited by the availability of other soil nutrients, especially P^38^ since symbiotic N fixation has a high demand of soil P^40^. Lower P (0.03 mM) reduced cell division in the cortex and prenodules and emerging nodules number as compared with medium P concentration (0.1 mM)^42^. The soil we used was low in P and therefore likely prevented a response in nitrogen fixation as elevated CO_2_ levels. Future studies are therefore needed to test the interactive effect of elevated CO_2_ and N deposition on N-fixing and non-N fixing plants in the absence of other nutrient limitations (e.g., P).

Overall, our study found that increasing N level could disadvantage N fixers relative to non-N fixers. Although actinorhizal plants can reduce nitrogen fixation in response to high soil N, this does not allow them to match the increasing growth of non-N fixers. This may help to explain the lack of N fixing plant dominance at present.

## Materials and methods

Three nitrogen fixing and three non-fixing plant species were grown at two levels of CO_2_ and four levels of N deposition. Three-month old seedlings of nitrogen fixers (*Alnus incana* ssp. *rugosa*, *Alnus viridis* ssp. *crispa*, and *Alnus rubra*) and the non-nitrogen fixers (*Betula pumila*, *Betula papyrifera*, *Betula glandulosa*) were grown in six growth chambers (Conviron A1000, Winnipeg, Canada), three of which were maintained at 400 ppm, and three of which were maintained at 1600 ppm CO_2_. There were 9 nitrogen fixers and 9 non-nitrogen fixers per each combination of N fertilization and CO_2_ level. In total, there were 72 plants in each level of CO_2_ and 36 plants in each level of N fertilization. In each chamber, the concentration of CO_2_ was monitored continuous and adjusted by injecting CO_2_ into the chambers as needed using a microcontroller-based system^43^. Each growth chamber had a 1000 L volume, and was set at 24 °C during the day and 19 °C during the night. The species are all in the Betulaceae family. *Betula* spp. are the closest relative to *Alnus* spp., and possibly still retain the precursor state of nitrogen fixation^44^. The plants were grown in *ca.* 0.5 L pots (6.4 cm diameter, D40H Deepots, Steuwe and Sons, Oregon) in a sandy soil collected from a forest with *A. viridis* ssp. *crispa* growing in the understory of mature *Pinus banksiana* trees. Previous study has shown that the soil from the site has an inorganic N level of 10.2 ± 0.6 mg/kg, and an extractable phosphate level of 0.98 ± 0.33 mg/kg^45^. All of the plants, including the non-nitrogen fixers, were inoculated with 10 mL of *Frankia* strain CpI1. The strain CpI1was cultured for 4 months in the sterilized liquid nitrogen-free *Frankia* Defined Minimal Medium (FDM) containing sodium succinate as carbon source^46^. The cultures were dispersed with a tissue grinder and then diluted 10 times in sterilized distilled water, using for inoculation^47^. Six weeks after inoculation, plants were fertilized with one of four levels of N fertilization (0, 0.42, 2.28, 8.4 mM ammonium nitrate) once a week for a total of 9 weeks. These four N fertilization treatments simulate a range of N deposition levels (0, 10, 50, and 200 kg N ha^−1^ year^−1^), as per Skogen^24^. A modified Rorison nutrient solution^26^ with the N concentration varied was used in the first fertilization. For the remaining 8 weeks plants received different levels of ammonium nitrate. After 9 weeks fertilization, plants were inoculated again with crushed nodules (3 mg/plant) of *Alnus*^47^. At week 24, Nitrogen fixation rate was estimated using the acetylene reduction assay^26^. Fresh nodules were placed into 50 mL bottles and sealed with a rubber septum with a 10% acetylene atmosphere. The bottles were incubated for 1 h at 22 ºC. A 5 mL gas sample from each bottle was collected and analyzed for ethylene with gas chromatograph (Varian 450) with a Haysep T column, FID and gas-sampling valve. The specific nodule activity (SNA) was expressed as the ethylene production rate per nodule dry mass. Nitrogen fixation rate per total plant mass was also calculated. Nodule allocation was calculated as the percentage of total plant dry mass.

## Statistics analysis

When analyzing the plant biomass, the data analysis used a three-factor least square model with CO_2_ level, N deposition level, and plant group (N fixers or non-N fixers), as well as their interaction, with species nested within plant group. When analyzing the nodulation allocation and nitrogen fixation, non-N fixers were excluded, the data analysis used a three-factor least square model with CO_2_ level, N deposition level, CO_2_ and N interaction, and N fixing species. The N deposition treatment was considered as a continuous variable, and the CO_2_ level and plant group were considered nominal variables. When there was a significant interaction between N deposition and the CO_2_ level or plant group, the effect of N deposition on the response variables was modelled using linear regressions, with the data transformed as needed in order to maintain homogeneity of variances. The data was analyzed using JMP Pro 14 (SAS Institute).

## Supplementary Information

Supplementary Information.
