# Supplementary material for: Ancient CO2 levels favor nitrogen fixing plants over a broader range of soil N compared to present
Source: Sci Rep. 2021 Feb 4;11:3038. doi: 10.1038/s41598-021-82701-7 (PMC7862480; doi:10.1038/s41598-021-82701-7)
Supplement: Supplementary file 1 — Supplementary Information. [file 41598_2021_82701_MOESM1_ESM.pdf]

Ancient CO_2 levels favor nitrogen fixing plants over a broader range of soil N compared to present

Haoran Chen* and John Markham

Department of Biological Sciences, University of Manitoba, Winnipeg, Canada

*Corresponding author: Email: chenh318@myumanitoba.ca, Tell: 2048072068

These authors contributed equally to this work

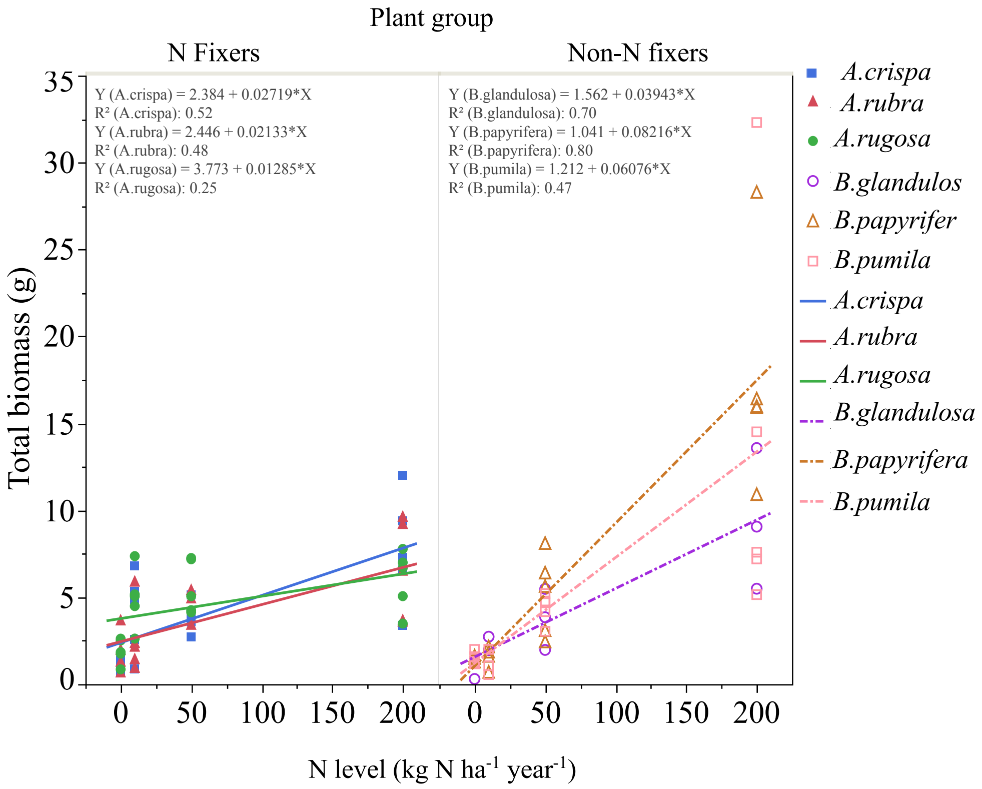

Supplementary Figure S1: Least squares fits of nitrogen fixing and non-nitrogen fixing plant species biomass versus soil level of N averaged by ambient and ancient CO_2 levels.

Supplementary Table S1: Three-factor least square model summarizing the effect of CO_2, N fertilization, plant group (N fixers/non-N fixers), as well as their interaction on plant biomass, with species nested within two plant groups (N fixers/non-N fixers). Bold indicates statistically significant effects (P < 0.05).

	Plant biomass	
	F ratio	P
Group	2.11	0.14
CO_2	1.51	0.30
Group*CO_2	0.95	0.33
N	100.12	**<0.0001**
Group*N	28.22	**<0.0001**
CO_2*N	8.03	**0.006**
Group*CO_2*N	0.61	0.44
Species [Group]	1.55	0.20

Supplementary Table S2: Three-factor least square model summarizing the effect of CO_2, N fertilization, and their interaction, and N fixing species on nodule allocation, specific nodule activity (SNA), and nitrogenase activity per plant mass. Bold indicates statistically significant effects ($P < 0.05$).

	Nodule allocation		Specific nodule activity (SNA)		Nitrogenase activity per plant mass	
	F ratio	P	F ratio	P	F ratio	P
CO_2	0.05	0.84	0.27	0.64	0.009	0.93
N	20.99	**<0.0001**	5.61	**0.02**	20.98	**<0.0001**
CO_2*N	0.0001	0.99	0.02	0.89	0.06	0.81
Species	3.35	**0.04**	1.91	0.16	3.64	**0.03**
